# Fasting plasma glucose trajectories are associated with ischemic stroke in the elderly: a longitudinal study using group-based trajectory modeling in Chinese communities

**DOI:** 10.1186/s12889-025-25773-8

**Published:** 2025-11-27

**Authors:** Qingwen Zhao, Ling Zhang, Xia Jiang, Xingyue Li, Xin Chen, Tianpei Ma, Xinyang Dui, Liang Lv, Xingyu Zhang, Haiyu Yan, Wanting Feng, Huifang Yang, Jinyu Xiao, Lu Long, Jiaqiang Liao, Tao Zhang, Yuqin Yao, Jiayuan Li

**Affiliations:** 1 Department of Epidemiology and Health Statistics, West China School of Public Health and West China Fourth Hospital, Sichuan University, Chengdu, China; 2 Department of Nutrition and Food Hygiene, West China School of Public Health and West China Fourth Hospital, Sichuan University, Chengdu, China; 3 Department of Occupational and Environmental Health, West China School of Public Health and West China Fourth Hospital, Sichuan University, Chengdu, China

**Keywords:** Stroke, Elderly population, Fasting plasma glucose, Ischemic stroke

## Abstract

**Background:**

Ischemic stroke (IS) has emerged as a severe health concern, particularly among the elderly. Although baseline fasting plasma glucose (FPG) has been linked to IS, research on the relationship between longitudinal FPG and IS risk is scarce. We aimed to investigate the association between FPG trajectories and IS incidence among the elderly using group-based trajectory modeling (GBTM).

**Methods:**

This longitudinal study in China enrolled 9,426 elderly individuals aged 65 and above, who had participated in health check-ups from 2017 to 2022. Employing a multivariable Cox proportional hazards regression model, we investigated the link between baseline FPG and IS incidence. GBTM was used to identify FPG trajectory patterns from the longitudinal data, which were then correlated with IS risk through further multivariable Cox regression analysis. We also performed stratified analyses and sensitivity analyses to explore these associations.

**Results:**

Diabetes FPG level in elderly individuals was significantly associated with an increased risk of IS at baseline, as indicated by both WHO criteria and ADA criteria. Longitudinal analysis revealed that individuals in the moderate increasing group and high stable group had 1.28 times (*HR* = 1.28, 95% CI 1.01‒1.62, *P* < 0.05) and 1.60 times (*HR* = 1.60, 95% CI 1.20‒2.15, *P* < 0.05) higher risks of IS, respectively, compared to the low stable group.

**Conclusions:**

Regular health screenings should emphasize monitoring FPG trends among the elderly to aid in IS prevention. Public health efforts targeting impaired fasting glucose control may reduce the risk of IS.

**Supplementary Information:**

The online version contains supplementary material available at 10.1186/s12889-025-25773-8.

## Background

Stroke represents high incidence, prevalence, mortality, and disability rates in China and has become a leading contributor to the national disease burden in recent years [1]. Ischemic stroke (IS), the predominant subtype, accounts for approximately 80% of all stroke cases [2]. From 1990 to 2019, the age-standardized mortality rate of IS in China showed no significant decline [3]. With the rapid aging of the population, implementing effective prevention strategies becomes crucial for reducing the disease burden of IS in China, especially among the elderly.

Fasting plasma glucose (FPG), an easily obtainable and cost-effective indicator of glucose metabolism, is routinely measured in basic public health services for the elderly. Elevated FPG can increase the level of circulating advanced glycation end products (AGEs), which may trigger inflammation and apoptosis in the endothelium and thus play a crucial role in plaque formation and atherosclerosis [4, 5]. These processes substantially contribute to cardiovascular diseases, including stroke [6].

Recent studies suggest the diabetes FPG level increases the risk of IS [7, 8], implying that maintaining FPG below the diabetes level could prevent IS. However, most of the epidemiological studies related to this topic rely on single baseline measurement, limiting our understanding of the long-term relationship between FPG and IS. Moreover, the optimal FPG for IS prevention remains debated [8–10]. Some evidence has linked impaired fasting glucose (IFG), a level below the diabetes threshold, to an increased risk of recurrent stroke or mortality in patients with IS [11]. Since IFG often progresses toward diabetes over time, its dynamic changes may also influence IS risk [9], but this relationship remains unclear. In addition, most prior studies have focused on general populations rather than the elderly, who may have a higher incidence of IS [12].

Therefore, this study aimed to: (1) examine the association between baseline FPG and the incidence of IS in older adults using both WHO and ADA criteria, and (2) investigate the relationship between long-term FPG trajectories and IS risk using group-based trajectory modeling (GBTM). The results of this study might provide evidence for more precise and effective public health strategies in preventing IS among the elderly.

## Research design and methods

### Detail of study population

This retrospective multi-community study primarily targeted Chinese elderly adults aged over 60 years who completed at least one basic public health physical examination at the Hongguang Community Health Service Center of Chengdu between 2017 and 2022. The study comprised 12,577 elderly individuals from 14 communities, representing 47.43% of the age-eligible local population. Sichuan Provincial Health Information Center matched health examination data with the disease diagnosis information system on the first page of each participant's medical records to obtain the first confirmed disease diagnosis information of participants from 2017.

**Fig. 1:**
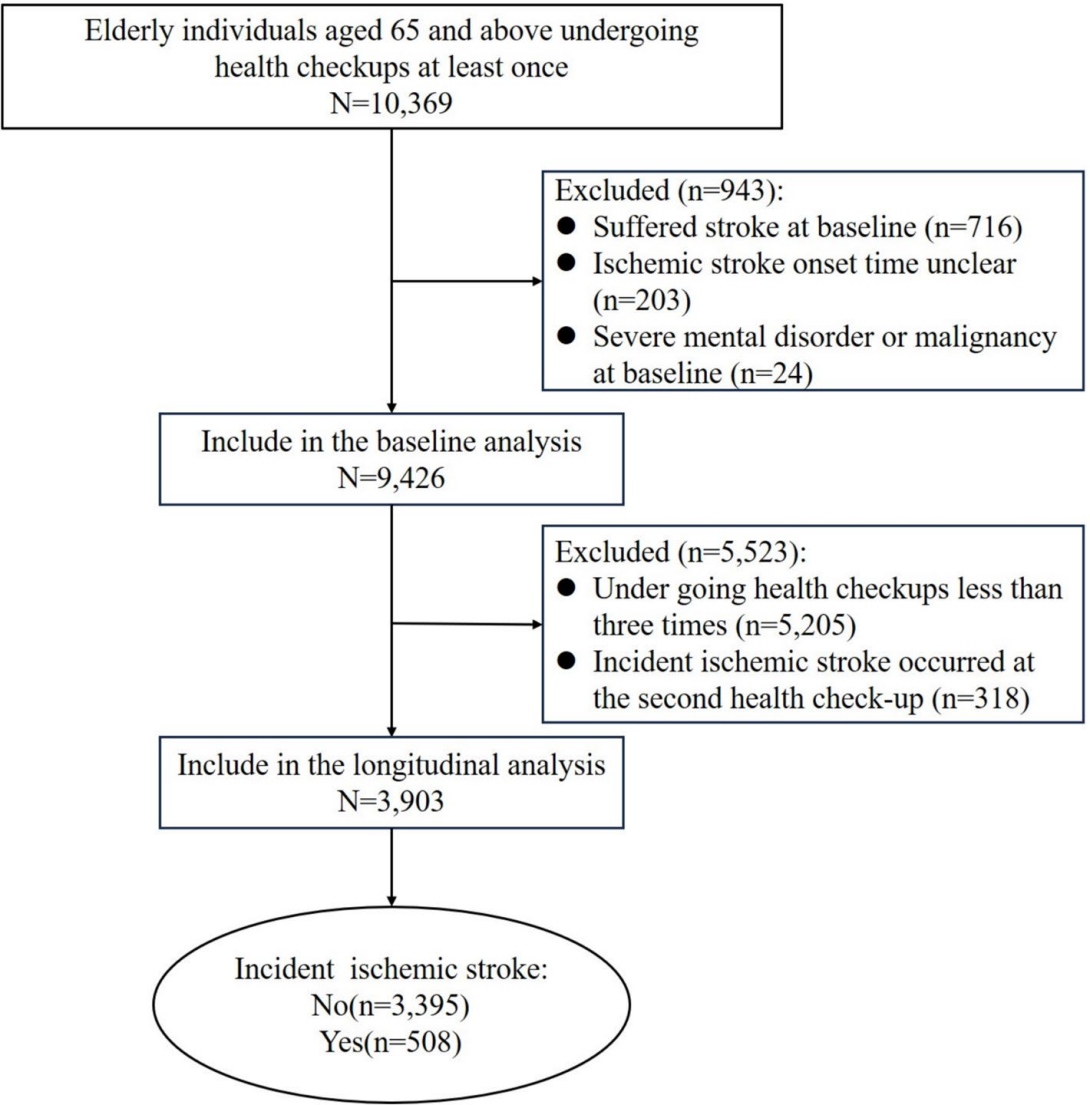
Flowchart of the study participants

We included the elderly aged 65 and above (*n* = 10,369) who completed baseline assessments. Individuals with a history of stroke at baseline (*n* = 716) or with unclear stroke onset (*n* = 203) were excluded to ensure accurate temporal assessment of incident IS. Participants with severe mental disorders or malignant tumors at baseline (*n* = 24) were further excluded due to potential impacts on glycemic metabolism. Ultimately, 9,426 individuals were included in the analysis of baseline FPG and IS risk. For the longitudinal trajectory analysis, we further excluded participants with fewer than three FPG measurements (*n* = 5,205) to ensure trajectory stability, and those who experienced IS before the second health examination (*n* = 318), leaving 3,903 participants (Fig. 1).

### Data collection

Health examination data included demographic information (age, gender, marital status, education level, etc.), general health examinations (height, weight, waist circumference, FPG, triglyceride (TG), total cholesterol (TC), diastolic blood pressure (DBP), systolic blood pressure (SBP), low-density lipoprotein cholesterol (LDL-c), high-density lipoprotein cholesterol (HDL-c), serum creatinine, etc.), lifestyle factors (exercise, diet, smoking status, drinking status, etc.), health status (hypertension history, diabetes history), and health assessments (health self-appraisal, etc.). The examinees fasted for 8–10 h overnight before venous blood was drawn the next morning during the physical examination. Blood samples were promptly sent for laboratory testing of various blood biochemical indicators.

### Definitions

Stroke was identified using International Classification of Diseases 10th Revision (ICD-10) codes I60-I64 [13], while IS was identified with ICD-10 codes I63 [14]. The incidence of IS was determined by the date that Sichuan Provincial Health Information Center matched.

We explored the association of baseline FPG groups with IS using two criteria. According to the World Health Organization (WHO) and Chinese clinical guidelines, FPG levels are categorized as normal (< 6.1 mmol/L), IFG (6.1 to 7.0 mmol/L), and diabetes level (≥ 7.0 mmol/L) [15, 16]. The American Diabetes Association (ADA) defined normal FPG as below 5.6 mmol/L, IFG as 5.6 to 7.0 mmol/L, and diabetes level as ≥ 7.0 mmol/L [17].

Body mass index (BMI) was defined as weight (kg)/height (m)^2^. Waist-to-height ratio (WHtR) was defined as waist (cm)/height (cm). Hypertension history was assessed using ICD-10 codes I10, and type 2 diabetes history with codes E11 [18]. BMI categories followed the Expert Consensus on Obesity Prevention and Treatment in China: normal weight (< 24 kg/m^2^), overweight (24 to 28 kg/m^2^), and obese (≥ 28 kg/m^2^) [19].

### Statistical analyses

Continuous variables were presented as means ± standard deviations (mean ± SD) for approximately normally distributed data and compared using t-tests. Skewed continuous variables (TC, HDL-c, LDL-c and TG) were summarized as medians (IQRs) and compared using the Wilcoxon rank-sum test. Categorical variables were expressed as frequencies and percentages (%) and compared using the chi-square test. Missing data for all covariates were less than 5% or none. To minimize potential bias, random multiple imputation was performed using the R package *mice* [20].

The proportional hazards (PH) assumption was assessed using Schoenfeld residuals for all Cox regression models. In the baseline analysis, the global Schoenfeld test indicated a violation of the PH assumption (*P* < 0.05), mainly due to the covariate age. To address this, an age-stratified Cox model was fitted [21, 22], with participants stratified into ≤ 70 years and > 70 years (based on the mean age). After stratification, the PH assumption was satisfied (*P* > 0.05). Hazard ratio (*HR*) and 95% confidence intervals (95% CI) were estimated after adjusting for age, gender, education level, waist-to-height ratio (WHtR), smoking, drinking, exercise, baseline SBP and DBP. Kaplan–Meier curves and log-rank tests were used to compare IS incidence across baseline FPG groups. A restricted cubic spline model, with 6.1 mmol/L as the FPG reference value, was applied to explore potential nonlinearity between FPG and IS, selecting three knots based on the smallest Akaike Information Criterion (AIC).

GBTM was then applied to identify distinct FPG trajectories representing similar longitudinal patterns. The optimal number of trajectories (ranging from 2 to 6) was determined using Bayesian Information Criterion (BIC), entropy, and average posterior probability [23, 24]. The final model was selected based on: (1) the lowest absolute BIC value, (2) the proportion of group distribution obtained from posterior probabilities of group members (P_j_) is no less than 5%, meaning that at least 5% of the participants within each trajectory group, and (3) an average posterior probability ≥ 0.7.

Additionally, Cox proportional hazards models were used to explore the association between FPG trajectories and incident IS. The PH assumption was satisfied (global Schoenfeld test *P* > 0.05). Model 1 was unadjusted; Model 2 adjusted for age and gender; and Model 3 additionally included WHtR, education, drinking, smoking, exercise, and baseline SBP and DBP. Age (≤ 70 *vs.* > 70 years), BMI (< 24 *vs.* ≥ 24 kg/m^2^) and gender (Male *vs.* Female) stratified analyses were also conducted to ensure robustness and comparability with the baseline analysis. Interaction tests were performed to assess the statistical significance of interactions between each potential effect modifier and trajectory groups. Besides, Model fit was evaluated using the AIC, with detailed AIC values presented in Supplementary Tables S4 and S8.

The *E-value* was employed to quantify the minimum strength of association that an unmeasured confounder would need to have with both the exposure and the outcome, conditional on the measured covariates, to fully account for the observed exposure–outcome relationship. An *E-value* of 1 indicates that no unmeasured confounding is required to explain the association, whereas larger *E-values* suggest that increasingly stronger confounder–exposure and confounder–outcome associations would be needed [25].

The data analysis process was conducted using R 4.3.3 version and SAS 9.4 version. We reported two-sided *P*-values, and *P* < 0.05 was considered statistically significant.

## Results

### Characteristics of included study participants

The primary analysis enrolled 9,426 participants (mean age 70.04 ± 5.27 years, 53.9% females), accruing 28,206 person-years of follow-up. The baseline characteristics of included and excluded participants were compared (Table S1). Of these included participants, 897 IS cases were documented. Significant differences in age, gender, education, WHtR, hypertension and diabetes history, smoking, drinking, exercise, FPG, FPG groups, DBP, and Pulse Pressure (PP) were observed between IS cases and non-cases (all *P* < 0.05, Table 1).

**Table 1:** Baseline characteristics of participants

Variables	Total (*N* = 9426)	Stroke		*P*
		**No (*n* = 8529)**	**Yes (*n* = 897)**	
Age (year)	70.04 ± 5.27	69.83 ± 5.19	72.10 ± 5.62	< 0.001
Gender, n (%)				0.008
Female	5076(53.9)	4555 (53.4)	521 (58.1)	
Male	4350 (46.1)	3974 (46.6)	376 (41.9)	
Education, n (%)				< 0.001
Primary school or below	5802 (61.5)	5186 (60.8)	616 (68.7)	
Junior high school	2663 (28.3)	2436 (28.6)	227 (25.3)	
Senior high school or above	961 (10.2)	907 (10.6)	54 (6.0)	
BMI (kg/m^2^)	24.05 ± 3.21	24.05 ± 3.18	24.11 ± 3.44	0.602
BMI groups, n(%)				0.217
Normal (< 24 kg/m^2^)	4824 (51.2)	4383 (51.4)	441 (49.2)	
Overweight or Obesity (> = 24 kg/m^2^)	4602 (48.8)	4146 (48.6)	456 (50.8)	
WHtR	0.53 ± 0.06	0.53 ± 0.06	0.54 ± 0.06	< 0.001
History of hypertension, n (%)				< 0.001
No	6334 (67.2)	5869 (68.8)	465 (51.8)	
Yes	3092 (32.8)	2660 (31.2)	432 (48.2)	
History of diabetes, n (%)				< 0.001
No	8249 (87.5)	7526 (88.2)	723 (80.6)	
Yes	1177 (12.5)	1003 (11.8)	1 74 (19.4)	
Smoking status, n (%)				< 0.001
No	6614 (70.2)	5929 (69.5)	685 (76.4)	
Yes	2812 (29.8)	2600 (30.5)	212 (23.6)	
Drinking status, n (%)				< 0.001
No	6130 (65.0)	5462 (64.0)	668 (74.5)	
Yes	3296 (35.0)	3067 (36.0)	229 (25.5)	
Lack of exercise, n (%)				0.046
No	7926 (84.1)	7193 (84.3)	733 (81.7)	
Yes	1500 (15.9)	1336 (15.7)	164 (18.3)	
FPG (mmol/L)	5.51 ± 1.28	5.50 ± 1.25	5.60 ± 1.55	0.031
FPG groups, n (%)				0.001
Normal FPG group (FPG < 6.1)	7565 (80.3)	6874 (80.6)	691 (77.0)	
IFG group (6.1 < = FPG < 7.0)	937 (9.9)	851 (10.0)	86 (9.6)	
Diabetes level group (FPG >= 7.0)	924 (9.8)	804 (9.5)	120 (13.4)	
LDL-c (mmol/L)	2.77 [2.27, 3.27]	2.78 [2.28, 3.28]	2.68 [2.19, 3.16]	0.001
HDL-c (mmol/L)	1.49 [1.27, 1.75]	1.49 [1.28, 1.76]	1.44 [1.24, 1.65]	< 0.001
TC (mmol/L)	5.13 [4.49, 5.81]	5.14 [4.50, 5.83]	5.03 [4.36, 5.65]	< 0.001
TG (mmol/L)	1.30 [0.96, 1.84]	1.30 [0.95, 1.83]	1.35 [0.98, 1.89]	0.068
SBP (mmHg)	139.96 ± 18.27	139.86 ± 18.26	141.00 ± 18.36	0.074
DBP (mmHg)	83.35 ± 10.94	83.42 ± 10.99	82.63 ± 10.41	0.038
PP (mmHg)	56.62 ± 13.33	56.43 ± 13.26	58.37 ± 13.88	< 0.001

*Abbreviation*: *BMI* body mass index, *WHtR* waist-to-height ratio, *FPG* fasting plasma glucose, *LDL-c* low-density lipoprotein cholesterol, *HDL-c* high-density lipoprotein cholesterol, *TC* total cholesterol, *TG* triglyceride, *SBP* Systolic Blood Pressure, *DBP* Diastolic Blood Pressure, *PP* Pulse Pressure

### Baseline FPG with the incident IS

The Kaplan–Meier curve and log-rank test showed significant differences among those baseline groups for IS survival probability (Figure S1).

For participants aged ≤ 70 years, each 1 mmol/L increase in FPG was associated with a 10% higher risk of IS (*HR* = 1.10, 95% CI 1.04–1.17, *P* < 0.05), showing no evidence of nonlinearity. In those aged > 70 years, IS risk increased by 7% per 1 mmol/L FPG (*HR* = 1.07, 95% CI 1.01–1.15, *P* < 0.05), showing a significant nonlinear association (*P*_non-linear_ < 0.05), with risk rising progressively at FPG levels above 6.1 mmol/L (Figure S2).

Using WHO criteria, participants with diabetes FPG level had a 1.51-fold higher IS risk in the ≤ 70 years age group (*HR* = 1.51, 95% CI 1.13–2.01, *P* < 0.05) and a 1.56-fold higher risk in those > 70 years (*HR* = 1.56, 95% CI 1.19–2.04, *P* < 0.05). In contrast, IFG was not significantly associated with IS in either age group. Similar patterns were observed under ADA criteria, with *HRs* of 1.51 (95% CI 1.13–2.02) and 1.58 (95% CI 1.20–2.09) for diabetes FPG level, while IFG remained nonsignificant (Tables S2–S3).

### Trajectories of FPG and the Kaplan–Meier curve

After excluding participants with fewer than three check-ups and incident IS before the second examination, the trajectory analysis included 3,903 individuals, among whom 508 developed IS.

**Fig. 2:**
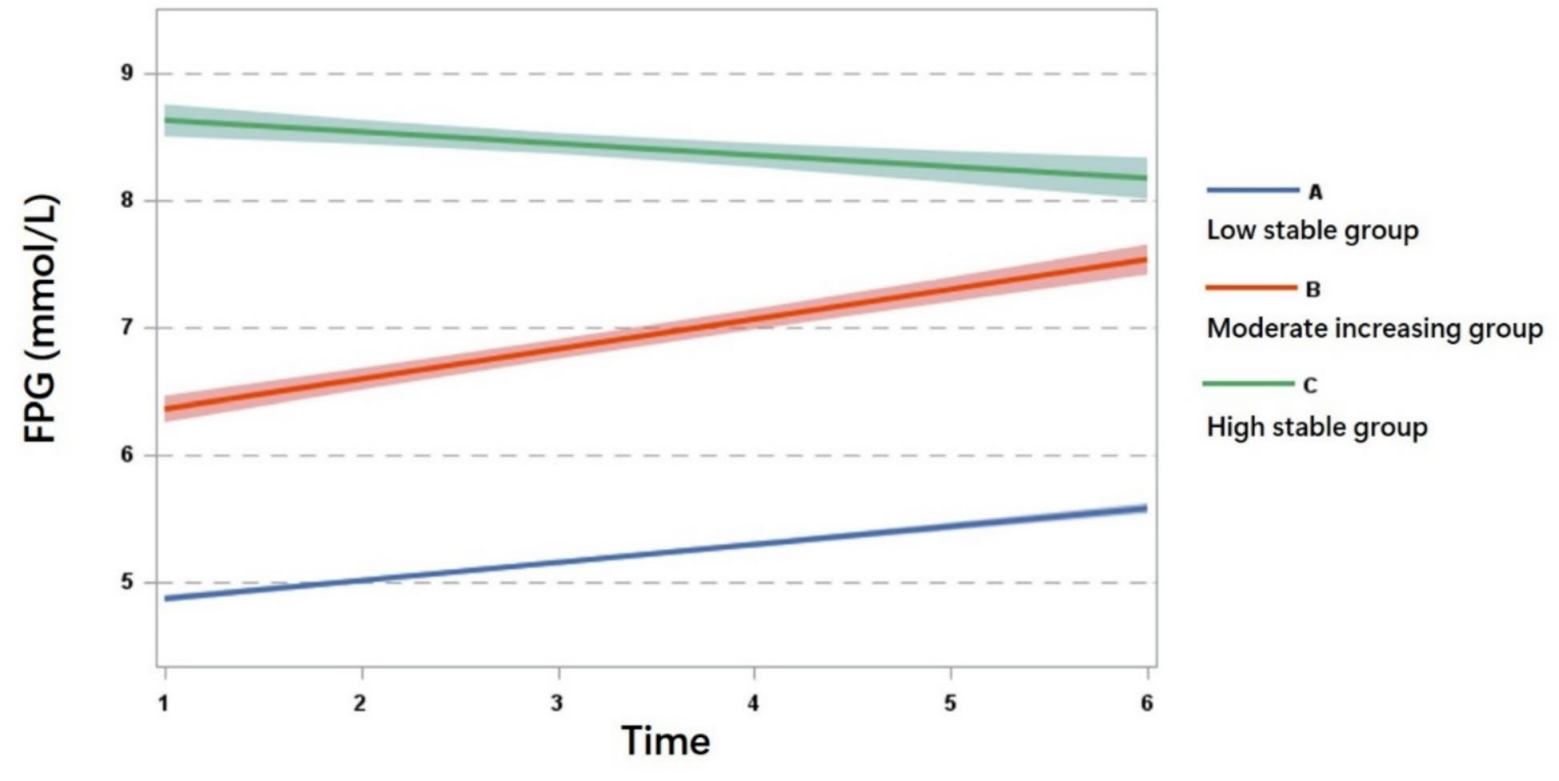
Long-term FPG trajectories derived from Group-Based trajectory modeling

Using GBTM, we identified three distinct trajectories of FPG (Table S5). Subgroup 1 was named “low stable group” (*N* = 3,044, P_j_ = 77.99%) as the trajectory maintained a low and stable FPG level over time. Subgroup 2 was named “moderate increasing group” (*N* = 561, P_j_ = 14.37%), showing a gradual increase in FPG from IFG to diabetes level. Subgroup 3 was named “high stable group” (*N* = 298, P_j_ = 7.64%) as it consistently exhibited FPG at diabetic level throughout (Fig. 2).

The average baseline FPG for the low stable, moderate increasing, and high stable groups was 4.90 ± 0.68, 6.37 ± 1.17, and 8.64 ± 1.85 mmol/L, respectively, with statistically significant differences across groups (*P* < 0.05) (Table S6). To better illustrate the characteristics of each trajectory, we also plotted box plots to show the P25-P75 ranges for each trajectory group at each time point (Figure S3).

**Fig. 3:**
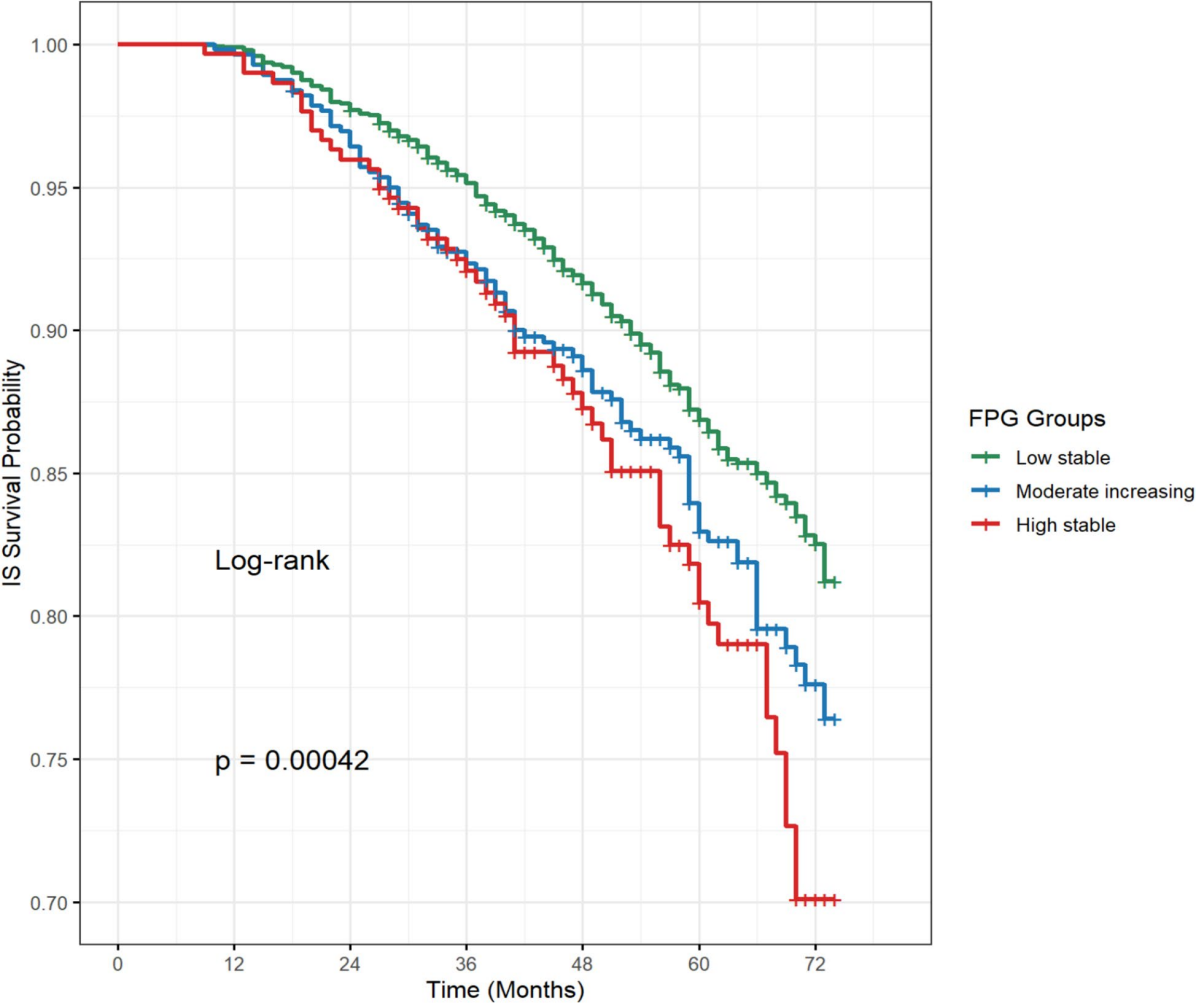
The Kaplan–Meier curve between long-term FPG trajectories and IS

The Kaplan–Meier curves showed steeper declines at each time point for the moderate increasing group and high stable group compared to the low stable group, indicating a correlation between higher FPG trajectories and a decreased survival probability for IS. The log-rank test confirmed significant differences among the three groups (Fig. 3). The incidence of IS increased across the three trajectory groups, with the cumulative incidence of IS in the high stable and moderate increasing groups higher than that in the low stable group (Table S7).

### Association of FPG trajectory groups with IS incidence

The analysis revealed that the risks of IS in the moderate increasing group and high stable group were 1.28 (95% CI 1.01‒1.62, *P* < 0.05) and 1.60 (95% CI 1.20‒2.15, *P* < 0.05) times greater compared to the low stable group, all *P* values for trend were significant (*P* < 0.001) (Table 2).

**Table 2:** Associations between FPG trajectories and incidence of IS

FPG groups	*HR* (95%CI)			*E-value*^*b*^
	**Model 1**	**Model 2**	**Model 3**	
Low stable group	Reference	Reference	Reference	Reference
Moderate increasing group	**1.34(1.07,1.69)**^**a**^	**1.33(1.06,1.68) **^**a**^	**1.28(1.01,1.62)**^**a**^	1.88(1.13)
High stable group	**1.64(1.23,2.18)**^**a**^	**1.66(1.25,2.21) **^**a**^	**1.60(1.20,2.15)**^**a**^	2.59(1.68)
*P* for trend	< 0.001	< 0.001	< 0.001	

Model 1 was unadjusted. Model 2 adjusted for age and gender. Model 3 further adjusted for and education level, WHtR, drinking status, smoking status, exercise, SBP and DBP, on the basis of Model 2

^a^*P* < 0.05

^b^*E-value* and minimum effect (confidence interval closest to the null) based on *HR* estimates with model 3

### Stratified analysis and sensitivity analyses

In stratified analyses, a significantly elevated risk of IS was observed only in the high stable FPG trajectory group among participants with a BMI ≥ 24 kg/m^2^. No significant differences were observed in the longitudinal associations between FPG trajectory groups and IS among age groups or by gender (Table 3).

**Table 3:** Stratified analysis

	**n**	**Low stable group (*n* = 3142)**	**Moderate increasing group (*n* = 595)**	**High stable group (*n* = 295)**	***P* for interaction**
Age groups					0.505
Age ≤ 70	2428	Reference	1.31(0.94,1.83)	1.75(1.19,2.56)^a^	
Age > 70	1475	Reference	1.20(0.86,1.68)	1.37(0.87,2.17)	
BMI groups					0.048
BMI < 24 kg/m^2^	1876	Reference	1.42(0.98,2.04)	0.92(0.49,1.75)	
BMI ≥ 24 kg/m^2^	2027	Reference	1.19(0.87,1.61)	1.86(1.33,2.60)^a^	
Gender					0.395
Female	2208	Reference	1.24(0.92,1.67)	1.33(0.92,1.94)	
Male	1695	Reference	1.30(0.90,1.90)	2.16(1.36,3.44)^a^	

Model was adjusted for age, gender, education level, WHtR, drinking status, smoking status, exercise, SBP and DBP on the basis of Model 2

^a^*P* < 0.05

In sensitivity analysis, the E-values were seen in the moderate increasing group (1.88 with E-value closest to 1 equal to 1.13) and high stable group (2.59 with E-value closest to 1 equal to 1.68) (Table 2).

## Discussion

This study demonstrated that baseline diabetes FPG level was significantly associated with the incidence of IS under both WHO and ADA criteria. To further enhance our understanding of the relationship between long-term FPG trajectories and IS risk, we applied GBTM to six years of follow-up data from 3,903 older adults in Chinese communities, identifying three distinct FPG trajectories and, for the first time, examining their associations with IS incidence. Individuals in the moderate increasing group and high stable group had a greater risk of IS. These findings suggest that public health strategies should focus not only on single-time FPG measurements but also on long-term changes in FPG levels.

Our findings support a clear association between baseline FPG and the incidence of IS in older adults, particularly when FPG reaches the diabetes range, whereas IFG was not significantly associated with IS risk. This pattern aligns with a large Chinese cohort study of adults aged 35–74, which reported that diabetes FPG level increased stroke risk under both ADA and WHO classifications (ADA: *HR* = 1.68, 95% CI 1.24–2.27; WHO: *HR* = 1.62, 95% CI 1.21–2.13), while IFG did not [7]. Likewise, a dose–response meta-analysis of 18 cohort studies including over 2.5 million middle-aged and older participants demonstrated a positive, nonlinear association between FPG and stroke risk, with a markedly higher risk observed at the highest glucose levels [26]. However, other research has shown that IFG can progress to diabetes, affecting stroke risk [9]. A meta-analysis of 15 studies found that IFG did not significantly increase stroke risk when defined as 5.6–6.9 mmol/L (*HR* = 1.08, 95% CI 0.94–1.23), but showed a modest risk elevation when defined as 6.1–6.9 mmol/L (*HR* = 1.21, 95% CI 1.02–1.44) [27]. Differences among studies may reflect variations in age composition, glycemic thresholds, and analytical approaches. Moreover, the definition and clinical implications of IFG remain debated. Future research with a larger population and comprehensive measurements should examine FPG–IS associations across different age strata and establish population-specific FPG cut-offs to better guide precision prevention strategies.

FPG levels fluctuate over time and are affected by various physiological and lifestyle factors, making single measurements prone to error and insufficient to reflect long-term glucose status [28]. Since the adverse effects of elevated FPG on IS are chronic, capturing its dynamic changes over the years is essential. Using GBTM, a semi-parametric approach that classifies individuals based on repeated measurements [23], we identified three distinct FPG trajectories and observed a clear dose–response relationship, with participants in higher-level trajectories had elevated IS risk. Notably, elderly individuals in the moderate-increasing trajectory likely represent a subgroup whose FPG gradually rises from the IFG range and may eventually cross the diabetes threshold if unmanagedand then elevated IS risk. This finding underscores the limitations of relying solely on baseline FPG. Recently, some studies used variability independent of the mean (VIM) to reflect long-term glucose status [29, 30]. For instance, a Korean cohort study reported that higher quartiles of FPG VIM were associated with progressively increased stroke risk [29], aligning with our observation that the moderate-increasing trajectory carries elevated IS risk. While glucose variability captures fluctuation, it does not reveal individual longitudinal patterns. Studies applying GBTM to FPG have shown that low- and high-increasing trajectories over six years were linked to higher all-cause mortality compared with stable trajectory [31], supporting the notion that rising glucose‒even below diabetic thresholds‒can increase adverse outcomes. Clinically, these results suggest that public health and clinical strategies should incorporate repeated FPG monitoring to identify individuals at risk of progressive hyperglycemia in regular health check-ups. Future studies should leverage large cohorts with repeated FPG assessments to jointly examine glucose variability and trajectory patterns in relation to IS and other cardiovascular outcomes, enabling improved risk stratification and targeted preventive interventions.

We further examined whether the associations between longitudinal changes in FPG levels and IS differed by individual characteristics. We observed that in different BMI subgroups the IS risk differed, and notably, participants with overweight or obesity in the high stable FPG trajectory group faced a significantly higher risk. This observation could be explained by obesity may accelerate the impact of elevated FPG on cardiovascular disease risk [32]. Some previous studies reported that FPG levels were more strongly associated with IS risk among participants who were male, hypertension [31, 33]. Those inconsistencies may be attributed to the distinct characteristics of different study populations.

One potential mechanism linking FPG to IS may involve brain microvascular dysfunction caused by hyperglycemia [34], which has also been observed in individuals with prediabetes, suggesting that IFG may contribute to an increased risk of stroke [7, 35]. Persistently elevated FPG can impair endothelial function [4, 36, 37], increase oxidative stress [38, 39], induce β-cell dysfunction [40], and elevate reactive oxygen species (ROS) levels [4]. ROS elevation activates the protein kinase C pathway, promotes the formation of AGEs, depletes nitric oxide, and increases angiotensin II production, which causes vasoconstriction and promotes the development of atherosclerotic plaques [41]. Moreover, ROS also upregulate pro-inflammatory genes, promoting endothelial apoptosis and thrombosis, which play a pivotal role in the progression of IS [42]. Chronic hyperglycemia is also associated with insulin resistance, which further promotes vasoconstriction, inflammation, and a prothrombotic state [4, 43]. Therefore, adopting healthy lifestyle behaviors—such as regular physical activity, maintaining a normal BMI, and following a balanced diet—may improve glucose homeostasis, help control FPG, and potentially reduce IS risk [31].

Our study has several limitations to acknowledge. Firstly, we utilized GBTM to analyze longitudinal community data between health check-ups, but did not fully capture specific changes at intermediate points. Secondly, the study sample was limited to Sichuan Province, China, restricting the applicability to other populations. Thirdly, due to the special characteristics of participants in our study, we had a higher IS probability than other studies, this may be related to the elderly population with higher age, lower education levels, prevalent comorbidities, and aging bodily functions. Additionally, the lack of comprehensive covariate measurements limits full adjustment for factors influencing FPG and IS risk including the medication use among the elderly. Although we conducted sensitivity analyses using E-values to assess the robustness of associations against potential unmeasured confounding, residual confounding cannot be entirely ruled out and may have partially influenced the observed results. Lastly, we did not measure Glycated Hemoglobin (HbA1c) or conduct oral glucose tolerance tests (OGTT). Although FPG may not capture postprandial glucose effects like HbA1c and OGTT [44], it remains both easily obtainable and cost-effective in basic public health services in many developing countries, especially for large elderly populations. Due to these limitations, future studies with larger cohorts and extended follow-ups are needed to further explore these relationships.

## Conclusion

FPG was strongly associated with IS risk in the elderly, as indicated by both baseline and longitudinal data. In older adults, the moderate increasing trajectory and the high stable trajectory were linked to higher IS risks. Attention should be given not only to those with diabetic level but also to those with IFG in the elderly. These findings may assist healthcare providers in offering better guidance and developing effective strategies to prevent IS among this vulnerable group.

## Supplementary Information

Supplementary Material 1.

Supplementary Material 2.

## Data Availability

Due to licensing restrictions, raw data cannot be publicly shared, but derived data supporting the findings are available from the corresponding author by reasonable request.
