# Supplementary material for: Fasting plasma glucose trajectories are associated with ischemic stroke in the elderly: a longitudinal study using group-based trajectory modeling in Chinese communities
Source: BMC Public Health. 2025 Nov 27;25:4339. doi: 10.1186/s12889-025-25773-8 (PMC12751438; doi:10.1186/s12889-025-25773-8)
Supplement: Supplementary file 1 — Supplementary Material 1. [file 12889_2025_25773_MOESM1_ESM.docx]

**Supplementary materials**

**Catalogue**

[**Figure S1. KM curves of baseline FPG groups in age groups** 2](#_Toc213857818)

[**Figure S2. The non-linear analysis between baseline constant FPG and IS** 4](#_Toc213857819)

[**Figure S3. Box plots of FPG trajectory groups** 5](#_Toc213857820)

[**Table S1. Baseline characteristics of included and excluded participants** 6](#_Toc213857821)

[**Table S2.** **Association between baseline FPG with incidence of IS in aged 65 to 70 years old** 9](#_Toc213857822)

[**Table S3.** **Association between baseline FPG with incidence of IS in aged above 70 years old** 10](#_Toc213857823)

[**Table S4.** **Association between baseline FPG and incident IS with model fit assessed by AIC** 11](#_Toc213857824)

[**Table S5. Model evaluation and model selection for the FPG trajectory group** 12](#_Toc213857825)

[**Table S6. Baseline characteristics of FPG trajectory groups** 13](#_Toc213857826)

[**Table S7. IS incident cumulative rate of each trajectory group** 16](#_Toc213857827)

[**Table S8.** **Association between FPG trajectory and incident IS with model fit assessed by AIC** 17](#_Toc213857828)


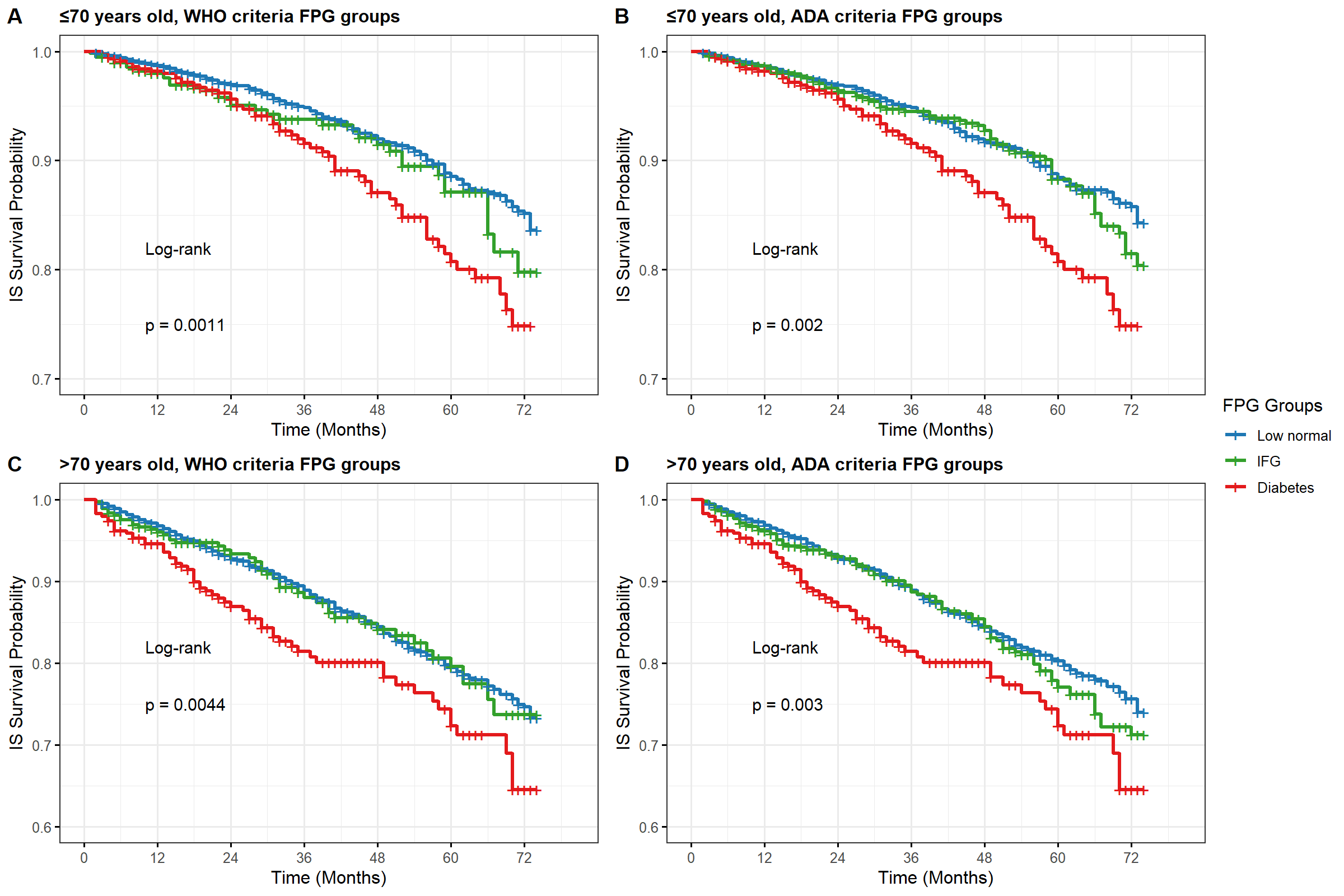


**Figure S1. KM curves of baseline FPG groups in age groups**

NOTES:

(A) Participants aged ≤70 years, IFG defined by WHO criteria (6.1–6.9mmol/L);

(B) Participants aged >70 years, IFG defined by WHO criteria (6.1–6.9mmol/L);

(C) Participants aged ≤70 years, IFG defined by ADA criteria (5.6–6.9mmol/L);

(D) Participants aged >70 years, IFG defined by ADA criteria (5.6–6.9mmol/L).


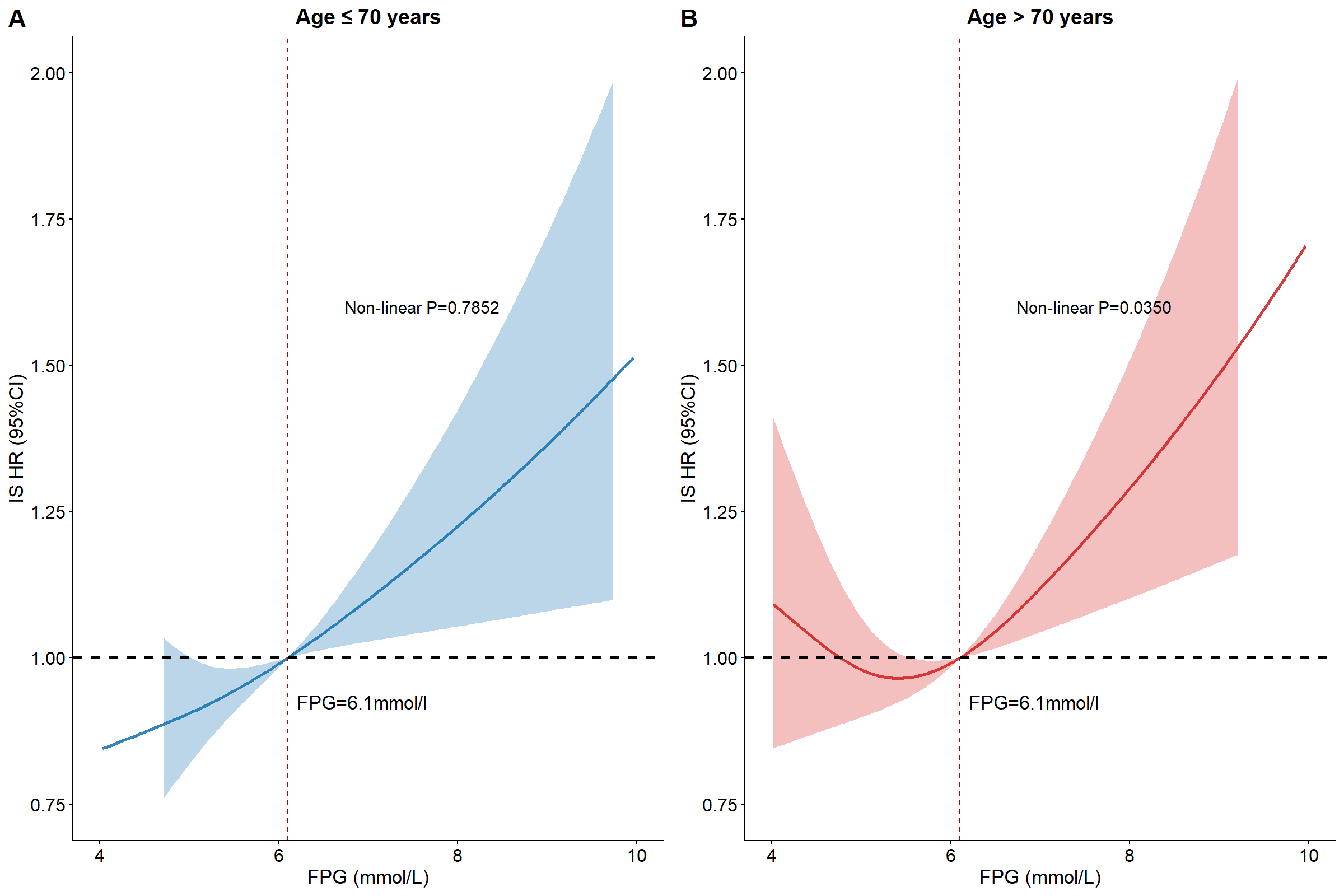


**Figure S2. The non-linear analysis between baseline constant FPG and IS**

NOTES:

(A) Participants aged ≤70 years;

(B) Participants aged >70 years.


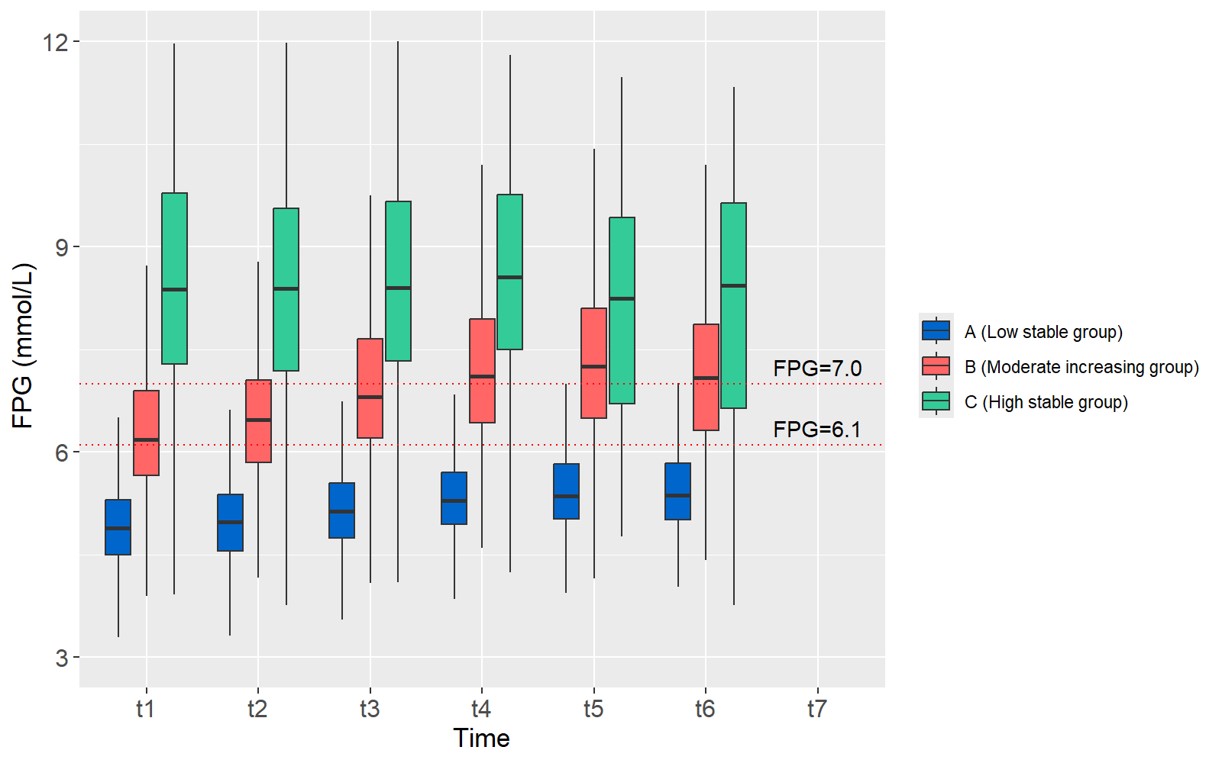


**Figure S3. Box plots of FPG trajectory groups**

**Table S1. Baseline characteristics of included and excluded participants**

| **Variables** | **Total**  **(N=10369)** | **Included** | | ***P*** |
| --- | --- | --- | --- | --- |
|  |  | **No (n=943)** | **Yes (n=9426)** |  |
| **Age (year)** | 70.29 (5.46) | 72.71 (6.55) | 70.04 (5.27) | <0.001 |
| **Gender, n (%)** |  |  |  | 0.049 |
| Female | 5616 (54.2) | 540 (57.3) | 5076 (53.9) |  |
| Male | 4753 (45.8) | 403 (42.7) | 4350 (46.1) |  |
| **Education, n=10354 (%)** |  |  |  | 0.266 |
| Primary school or below | 6358 (61.4) | 567 (60.2) | 5791 (61.5) |  |
| Junior high school | 2924 (28.2) | 263 (27.9) | 2661 (28.3) |  |
| Senior high school or above | 1072 (10.4) | 112 (11.9) | 960 (10.2) |  |
| **BMI (kg/m^2^)** | 24.08 (3.23) | 24.37 (3.38) | 24.05 (3.21) | 0.005 |
| **BMI groups, n (%)** |  |  |  | 0.021 |
| Normal (<24 kg/m^2^) | 5269 (50.8) | 445 (47.2) | 4824 (51.2) |  |
| Overweight or Obesity (>=24kg/m^2^) | 5100 (49.2) | 498 (52.8) | 4602 (48.8) |  |
| **WHtR** | 0.53 (0.06) | 0.54 (0.06) | 0.53 (0.06) | <0.001 |
| **History of hypertension，n (%)** |  |  |  | <0.001 |
| No | 6628 (63.9) | 294 (31.2) | 6334 (67.2) |  |
| Yes | 3741 (36.1) | 649 (68.8) | 3092 (32.8) |  |
| **History of diabetes, n (%)** |  |  |  | <0.001 |
| No | 8961 (86.4) | 712 (75.5) | 8249 (87.5) |  |
| Yes | 1408 (13.6) | 231 (24.5) | 1177 (12.5) |  |
| **Smoking status,**  **n =10355 (%)** |  |  |  | 0.345 |
| No | 7279 (70.3) | 676 (71.7) | 6603 (70.2) |  |
| Yes | 3076 (29.7) | 267 (28.3) | 2809 (29.8) |  |
| **Drinking status,**  **n=10353 (%)** |  |  |  | 0.001 |
| No | 6782 (65.5) | 664 (70.4) | 6118 (65.0) |  |
| Yes | 3571 (34.5) | 279 (29.6) | 3292 (35.0) |  |
| **Lack of exercise,**  **n=10356 (%)** |  |  |  | 0.86 |
| No | 8712 (84.1) | 794 (84.4) | 7918 (84.1) |  |
| Yes | 1644 (15.9) | 147 (15.6) | 1497 (15.9) |  |
| **FPG (mmol/L)** | 5.53 (1.29) | 5.71 (1.37) | 5.51 (1.28) | <0.001 |
| **FPG groups, n (%)** |  |  |  | <0.001 |
| Normal FPG group  (FPG< 6.1) | 8271 (79.8) | 706 (74.9) | 7565 (80.3) |  |
| IFG group (6.1<=FPG<7.0) | 1047 (10.1) | 110 (11.7) | 937 (9.9) |  |
| Diabetes level group (FPG>=7.0) | 1051 (10.1) | 127 (13.5) | 924 (9.8) |  |
| **LDL-c (mmol/L)** | 2.76 [2.26, 3.27] | 2.62 [2.04, 3.17] | 2.77 [2.28, 3.28] | <0.001 |
| **HDL-c (mmol/L)** | 1.48 [1.27, 1.75] | 1.47 [1.26, 1.74] | 1.49 [1.27, 1.75] | 0.103 |
| **TC (mmol/L)** | 5.11 [4.47, 5.80] | 4.95 [4.21, 5.69] | 5.13 [4.49, 5.81] | <0.001 |
| **TG (mmol/L)** | 1.30 [0.96, 1.84] | 1.29 [1.00, 1.85] | 1.30 [0.96, 1.84] | 0.185 |
| **SBP (mmHg)** | 140.01 (18.28) | 140.51 (18.43) | 139.96 (18.27) | 0.386 |
| **DBP (mmHg)** | 83.24 (10.96) | 82.16 (11.08) | 83.35 (10.94) | 0.001 |
| **PP (mmHg)** | 56.77 (13.39) | 58.35 (13.92) | 56.62 (13.33) | <0.001 |

**Abbreviation:** BMI, body mass index; WHtR, waist-to-height ratio; FPG, fasting plasma glucose; LDL-c, low-density lipoprotein cholesterol; HDL-c, high-density lipoprotein cholesterol; TC, total cholesterol; TG, triglyceride; SBP, Systolic Blood Pressure; DBP, Diastolic Blood Pressure; PP, Pulse Pressure.

* The excluded participants were generally older and had higher BMI, WHtR, and FPG levels, as well as a higher prevalence of hypertension and diabetes, but lower LDL-c, TC, and alcohol consumption. These differences are expected, as excluded individuals predominantly had pre-existing IS or metabolic disorders.

**Table S2.** **Association between baseline FPG with incidence of IS in aged 65 to 70 years old**

| **Variables** | **HR (95% CI)** | | | ***E-value*^d^** |
| --- | --- | --- | --- | --- |
|  | **Model 1** | **Model 2** | **Model 3** |  |
| **Constant FPG** | 1.12 (1.06,1.19) ^a^ | 1.11 (1.05,1.18) ^a^ | 1.10(1.04,1.17) ^a^ | 1.45(1.24) |
| **WHO^b^ criteria FPG groups** |  |  |  |  |
| Low normal FPG group | Reference | Reference | Reference | Reference |
| IFG group | 1.29(0.93,1.79) | 1.29 (0.93,1.79) | 1.25(0.90,1.74) | 1.80(1.00) |
| Diabetes group | 1.65(1.25,2.19) ^a^ | 1.58(1.19,2.09) ^a^ | 1.51(1.13,2.01) ^a^ | 2.38(1.52) |
| *P* for trend | <0.001 | <0.001 | 0.003 |  |
| **ADA^c^ criteria FPG groups** |  |  |  |  |
| Low normal FPG group | Reference | Reference | Reference | Reference |
| IFG group | 1.12(0.88,1.43) | 1.14(0.89,1.45) | 1.10(0.87,1.41) | 1.45(1.00) |
| Diabetes group | 1.66(1.25,2.20) ^a^ | 1.58(1.19,2.11) ^a^ | 1.51(1.13,2.02) ^a^ | 2.38(1.51) |
| *P* for trend | 0.001 | 0.002 | 0.009 |  |

Model 1 was unadjusted. Model 2 adjusted for age, gender. Model 3 further adjusted for education level, WHtR, drinking status, smoking status, exercise, baseline SBP and DBP on the basis of Model 2. ^a^*P*＜0.05.

^b^: WHO, World Health Organization, defines IFG as FPG of 6.1–6.9mmol/L.

^c^: ADA, the American Diabetes Association sets the IFG range at 5.6–6.9mmol/L.

^d^: *E-value* and minimum effect (confidence interval closest to the null) based on HR estimates with model 3.

**Table S3.** **Association between baseline FPG with incidence of IS in aged above 70 years old**

| **Variables** | **HR (95% CI)** | | | ***E-value*^d^** |
| --- | --- | --- | --- | --- |
|  | **Model 1** | **Model 2** | **Model 3** |  |
| **Constant FPG** | 1.08(1.01,1.15) ^a^ | 1.08(1.01,1.15) ^a^ | 1.07(1.01,1.15) ^a^ | 1.36(1.09) |
| **WHO^b^ criteria FPG groups** |  |  |  |  |
| Low normal FPG group | Reference | Reference | Reference | Reference |
| IFG group | 1.01(0.75,1.37) | 1.03(0.76,1.39) | 1.00(0.73,1.35) | 1.10(1.00) |
| Diabetes group | 1.56(1.19,2.04) ^a^ | 1.58(1.21,2.07) ^a^ | 1.56(1.19,2.04) ^a^ | 2.48(1.65) |
| *P* for trend | 0.004 | 0.003 | 0.005 |  |
| **ADA^c^ criteria FPG groups** |  |  |  |  |
| Low normal FPG group | Reference | Reference | Reference | Reference |
| IFG group | 1.11(0.89,1.38) | 1.10(0.88,1.37) | 1.07(0.85,1.33) | 1.33(1.00) |
| Diabetes group | 1.60(1.22,2.10) ^a^ | 1.61(1.23,2.12) ^a^ | 1.58(1.20,2.09) ^a^ | 2.54(1.69) |
| *P* for trend | 0.002 | 0.002 | 0.005 |  |

Model 1 was unadjusted. Model 2 adjusted for age and gender. Model 3 further adjusted for education level, WHtR, drinking status, smoking status, exercise, baseline SBP and DBP on the basis of Model 2. ^a^*P*＜0.05.

^b^: WHO, World Health Organization, defines IFG as FPG of 6.1–6.9mmol/L.

^c^: ADA, the American Diabetes Association sets the IFG range at 5.6–6.9mmol/L.

^d^: *E-value* and minimum effect (confidence interval closest to the null) based on HR estimates with model 3.

**Table S4.** **Association between baseline FPG and incident IS with model fit assessed by AIC**

| **Variables** | **HR (95% CI)** | | |
| --- | --- | --- | --- |
|  | **Model 1** | **Model 2** | **Model 3** |
| **Aged 65 to70 years old** |  |  |  |
| Constant FPG | 6475.981 | 6455.605 | **6450.149** |
| WHO^a^ criteria FPG groups | 6478.45 | 6458.484 | **6453.248** |
| ADA^b^ criteria FPG groups | 6479.69 | 6459.589 | **6454.223** |
| **Aged 65 to70 years old** |  |  |  |
| Constant FPG | 7309.729 | 7307.023 | **7302.163** |
| WHO^a^ criteria FPG groups | 7307.411 | 7304.545 | **7299.317** |
| ADA^b^ criteria FPG groups | 7306.633 | 7303.855 | **7299.009** |

**Abbreviation:** AIC, the Akaike Information Criterion

Model 1 was unadjusted. Model 2 adjusted for age and gender. Model 3 further adjusted for education level, WHtR, drinking status, smoking status, exercise, baseline SBP and DBP on the basis of Model 2.

^a^: WHO, World Health Organization, defines IFG as FPG of 6.1–6.9mmol/L.

^b^: ADA, the American Diabetes Association sets the IFG range at 5.6–6.9mmol/L.

Among the models, Model 3 always demonstrated the best fit by using AIC.

**Table S5. Model evaluation and model selection for the FPG trajectory group**

| **Group**  **(Subgroup order)** | ***Avepp(%)*** | ***OCC*** | ***P_j_(%)*** | ***π_j_(%)*** | ***BIC*** |
| --- | --- | --- | --- | --- | --- |
| ***1Group (3)*** | 100.00 | . | 100.00 | 100.00 |  |
| ***2Group (1 2)*** | 99.14-96.76 | 22.3-153.8 | 83.94-16.06 | 83.73-16.27 | 3816.07 |
| ***3Group (1 1 1)*** | **97.61-88.81-91.54** | **12.0-44.0-134.7** | **77.99-14.37-7.64** | **77.29-15.28-7.44** | **611.84** |
| ***4Group (1 1 1 2)*** | 97.09-87.17-89.99-90.98 | 10.8-38.6-121.4-377.0 | 76.43-14.01-6.97-2.59 | 75.53-14.97-6.89-2.61 | 326.34 |
| ***5Group (1 2 2 2 2)*** | 96.82-85.03-86.34-91.12-90.91 | 10.5-33.3-105.7-302.1-462.7 | 75.33-13.73-5.69-3.18-2.08 | 74.39-14.56-5.64-3.29-2.12 | 282.89 |
| ***6Group (1 2 2 2 2 2)*** | 78.00-94.32-81.02-84.72-90.42-91.63 | 18.7-7.3-63.3-141.7-351.1-535.2 | 14.42-71.18-6.10-3.72-2.61-1.97 | 15.97-69.33-6.32-3.77-2.62-2.01 | 153.47 |

**Abbreviation：**

AvePP, average posterior probability;

OCC, odds of correct classification;

P_j_: Proportion of group distribution obtained based on posterior probability of group members;

**π**_j_: Proportion of group distribution obtained based on probability of group members;

BIC, Bayesian information criterion.

**Table S6. Baseline characteristics of FPG trajectory groups**

| **Variables** | **Total**  **(N=3903)** | **Low stable group （n=3044）** | **Moderate increasing group （n=561）** | **High stable group （n=298）** | ***P*** |
| --- | --- | --- | --- | --- | --- |
| **Age (year)** | 70.04±4.82 | 70.02±4.89 | 70.27±4.68 | 69.78±4.34 | 0.330 |
| **Gender, n (%)** |  |  |  |  | 0.126 |
| Female | 2208 (56.6) | 1704 (56.0) | 319 (56.9) | 185 (62.1) |  |
| Male | 1695 (43.4) | 1340 (44.0) | 242 (43.1) | 113 (37.9) |  |
| **Education, n (%)** |  |  |  |  | 0.126 |
| Primary school or below | 2560 (65.6) | 2021 (66.4) | 362 (64.5) | 177 (59.4) |  |
| Junior high school | 1019 (26.1) | 770 (25.3) | 156 (27.8) | 93 (31.2) |  |
| Senior high school or above | 324 (8.3) | 253 (8.3) | 43 (7.7) | 28 (9.4) |  |
| **BMI (kg/m^2^)** | 24.30±3.21 | 24.05±3.17 | 25.09±3.16 | 25.36±3.24 | <0.001 |
| **BMI groups, n (%)** |  |  |  |  | <0.001 |
| Normal (<24 kg/m2) | 1876 (48.1) | 1569 (51.5) | 202 (36.0) | 105 (35.2) |  |
| Overweight or Obesity (>=24kg/m2) | 2027 (51.9) | 1475 (48.5) | 359 (64.0) | 193 (64.8) |  |
| **WHtR** | 0.54±0.06 | 0.53±0.06 | 0.55±0.06 | 0.56±0.06 | <0.001 |
| **Abnormal WHtR, n (%)** |  |  |  |  | <0.001 |
| No | 981 (25.1) | 838 (27.5) | 98 (17.5) | 45 (15.1) |  |
| Yes | 2922 (74.9) | 2206 (72.5) | 463 (82.5) | 253 (84.9) |  |
| **Central obesity, n (%)** |  |  |  |  | <0.001 |
| No | 2580 (66.1) | 2114 (69.4) | 310 (55.3) | 156 (52.3) |  |
| Yes | 1323 (33.9) | 930 (30.6) | 251 (44.7) | 142 (47.7) |  |
| **History of hypertension, n (%)** |  |  |  |  | <0.001 |
| No | 2411 (61.8) | 1984 (65.2) | 274 (48.8) | 153 (51.3) |  |
| Yes | 1492 (38.2) | 1060 (34.8) | 287 (51.2) | 145 (48.7) |  |
| **History of diabetes, n (%)** |  |  |  |  | <0.001 |
| No | 3361 (86.1) | 2954 (97.0) | 331 (59.0) | 76 (25.5) |  |
| Yes | 542 (13.9) | 90 (3.0) | 230 (41.0) | 222 (74.5) |  |
| **Smoking status, n (%)** |  |  |  |  | 0.966 |
| No | 2909 (74.5) | 2267 (74.5) | 418 (74.5) | 224 (75.2) |  |
| Yes | 994 (25.5) | 777 (25.5) | 143 (25.5) | 74 (24.8) |  |
| **Drinking status, n (%)** |  |  |  |  | 0.753 |
| No | 2745 (70.3) | 2135 (70.1) | 402 (71.7) | 208 (69.8) |  |
| Yes | 1158 (29.7) | 909 (29.9) | 159 (28.3) | 90 (30.2) |  |
| **Lack of exercise, n (%)** |  |  |  |  | 0.003 |
| No | 3214 (82.3) | 2474 (81.3) | 480 (85.6) | 260 (87.2) |  |
| Yes | 689 (17.7) | 570 (18.7) | 81 (14.4) | 38 (12.8) |  |
| **FPG (mmol/L)** | 5.40±1.40 | 4.90±0.68 | 6.37±1.17 | 8.64±1.85 | <0.001 |
| **FPG groups, n (%)** |  |  |  |  | <0.001 |
| Normal FPG group | 3192 (81.8) | 2922 (96.0) | 250 (44.6) | 20 (6.7) |  |
| IFG group | 313 (8.0) | 100 (3.3) | 179 (32.9) | 34 (11.4) |  |
| Diabetes level group | 398 (10.2) | 22 (0.7) | 132 (23.5) | 244 (81.9) |  |
| **LDL-c (mmol/L)** | 2.71 [2.25, 3.20] | 2.70 [2.24, 3.19] | 2.71 [2.22, 3.22] | 2.81 [2.23, 3.32] | 0.476 |
| **HDL-c (mmol/L)** | 1.42 [1.24, 1.65] | 1.44 [1.25, 1.67] | 1.35 [1.18, 1.57] | 1.36 [1.21, 1.56] | <0.001 |
| **TC (mmol/L)** | 5.07 [4.44, 5.71] | 5.07 [4.46, 5.71] | 5.02 [4.33, 5.65] | 5.08 [4.39, 5.86] | 0.223 |
| **TG (mmol/L)** | 1.32 [0.98, 1.86] | 1.27 [0.94, 1.77] | 1.54 [1.13, 2.09] | 1.58 [1.16, 2.26] | <0.001 |
| **SBP (mmHg)** | 139.19±17.31 | 138.32±17.16 | 141.52±17.18 | 143.67±18.03 | <0.001 |
| **DBP (mmHg)** | 82.23±10.10 | 82.09±10.08 | 82.76±10.21 | 82.65±10.14 | 0.268 |
| **PP (mmHg)** | 56.96±13.38 | 56.23±13.08 | 58.76±13.77 | 61.03±14.58 | <0.001 |
| **Ischemic stroke** |  |  |  |  | 0.001 |
| No | 3395 (87.0) | 2679 (88.0) | 472 (84.1) | 244 (81.9) |  |
| Yes | 508 (13.0) | 365 (12.0) | 89 (15.9) | 54 (18.1) |  |

**Abbreviation:**

BMI, body mass index; WHtR, waist-to-height ratio; FPG, fasting plasma glucose; LDL-c, low-density lipoprotein cholesterol; HDL-c, high-density lipoprotein cholesterol; TC, total cholesterol; TG, triglyceride; TyG index, triglyceride-Glucose; SBP, Systolic Blood Pressure; DBP, Diastolic Blood Pressure;PP, Pulse Pressure.

**Table S7. IS incident cumulative rate of each trajectory group**

|  | T1 (%) | T2 (%) | T3 (%) | T4 (%) | T5 (%) | T6 (%) |
| --- | --- | --- | --- | --- | --- | --- |
| Low stable group | 0.09 | 2.36 | 4.89 | 7.72 | 10.77 | 12.58 |
| Moderate increasing group | 0.35 | 3.74 | 7.66 | 10.69 | 14.082 | 16.04 |
| High stable group | 0.33 | 4.02 | 7.71 | 11.40 | 15.10 | 18.12 |

**Table S8.** **Association between FPG trajectory and incident IS with model fit assessed by AIC**

| **Variables** | **HR (95% CI)** | | |
| --- | --- | --- | --- |
|  | **Model 1** | **Model 2** | **Model 3** |
| AIC | 7962.756 | 7933.006 | 7936.707 |
| C index | 0.537 | 0.596 | 0.611 |

**Abbreviation:** AIC, the Akaike Information Criterion; C index, the concordance index.

Model 1 was unadjusted. Model 2 adjusted for age and gender. Model 3 further adjusted for education level, WHtR, drinking status, smoking status, exercise, baseline SBP and DBP on the basis of Model 2.

*Among the models, the AIC of Model 3 is slightly higher than that of Model 2. However, Model 3 includes additional covariates, which better capture the relevant clinical factors and provide a more comprehensive analysis of the relationship between FPG trajectories and IS. Moreover, the concordance index (C index) for Model 3 (0.611) is slightly higher than that of Model 2 (0.596), indicating that Model 3 demonstrates a better discriminatory ability in predicting incident IS. This suggests that the inclusion of additional covariates improves the model's performance in terms of classification accuracy. Importantly, the direction of the effect of the FPG trajectory groups remains consistent between Model 3 and Model 2. Therefore, we chose to report the effect of Model 3.
